# The *EIF2AK4/*rs4594236 AG/GG Genotype Is a Hazard Factor of Immunoglobulin Therapy Resistance in Southern Chinese Kawasaki Disease Patients

**DOI:** 10.3389/fgene.2022.868159

**Published:** 2022-06-22

**Authors:** Hongyan Yu, Fucheng Liu, Kaining Chen, Yufen Xu, Yishuai Wang, Lanyan Fu, Huazhong Zhou, Lei Pi, Di Che, Hehong Li, Xiaoqiong Gu

**Affiliations:** ^1^ Department of Clinical Biological Resource Bank, Guangdong Provincial Key Laboratory of Research in Structural Birth Defect Disease, Guangzhou Institute of Pediatrics, Guangzhou Women and Children’s Medical Center, Guangzhou Medical University, Guangzhou, China; ^2^ Department of Cardiology, The First Affiliated Hospital of Jinan University, Guangzhou, China; ^3^ Department of Radiology, Guangzhou Women and Children’s Medical Center, Guangzhou Medical University, Guangzhou, China; ^4^ Department of Blood Transfusion and Clinical Lab, Guangzhou Institute of Pediatrics, Guangzhou Women and Children’s Medical Center, Guangzhou Medical University, Guangzhou, China

**Keywords:** Kawasaki disease, IVIG resistance, polymorphism, *EIF2AK4*/rs4594236, intravenous immunoglobulin

## Abstract

**Background:** Kawasaki disease (KD) is an acute, self-limited vasculitis disorder of unknown etiology in children. Immunologic abnormalities were detected during the acute phase of KD, which reflected that the effect cells of the activated immune system markedly increased cytokine production. High-dose intravenous immunoglobulin (IVIG) therapy is effective in resolving inflammation from KD and reducing occurrence of coronary artery abnormalities. However, 10%–20% of KD patients have no response to IVIG therapy, who were defined as IVIG resistance. Furthermore, these patients have persistent inflammation and increased risk of developing coronary artery aneurysm (CAA). *EIF2AK4* is a stress sensor gene and can be activated by pathogen infection. In addition, the polymorphisms of *EIF2AK4* were associated with various blood vessel disorders. However, it remains unclear whether the *EIF2AK4* gene polymorphisms were related to IVIG therapy outcome in KD patients.

**Methods:** *EIF2AK4/*rs4594236 polymorphism was genotyped in 795 IVIG response KD patients and 234 IVIG resistant KD patients through TaqMan, a real-time polymerase chain reaction. The odds ratios (ORs) and 95% confidence intervals (CIs) were calculated to assess the strength of association between *EIF2AK4/*rs4594236 polymorphism and IVIG therapeutic effects.

**Results:** Our results showed that the *EIF2AK4*/rs4594236 AG/GG genotype was significantly associated with increased risk to IVIG resistance compared to the AA genotype (AG vs. AA: adjusted ORs = 1.71, 95% CIs = 1.17–2.51, and *p* = 0.0061; GG vs. AA: adjusted ORs = 2.09, 95% CIs = 1.36–3.23, and *p* = 0.0009; AG/GG vs. AA: adjusted ORs = 1.82, 95% CIs = 1.27–2.63, and *p* = 0.0013; and GG vs. AA/AG: adjusted ORs = 1.45, 95% CI = 1.04–2.02, and *p* = 0.0306). Furthermore, the stratified analysis of age and gender in the KD cohort indicated that male patients carrying the rs4594236 AG/GG genotype tends to be more resistant to IVIG therapy than female patients.

**Conclusion:** These results suggested that *EIF2AK4/*rs4594236 polymorphism might be associated with increased risk of IVIG resistance in southern Chinese KD patients.

## Introduction

Kawasaki disease (KD) is an acute, self-limited vasculitis disease in children aged from 6 months to 5 years (Burns and Glodé, 2004). Immunologic abnormalities were detected during the acute phase of KD, which reflected that the effect cells of the activated immune system markedly increased cytokine production (Burns and Glodé, 2004). The etiology of KD is unknown, while several epidemiological and clinical reports have suggested that KD might be triggered by infectious agents or viruses (Sharma et al., 2021). This was evidenced by the fact that proinflammatory cytokines (IL-6, IL-10, TNFα, and IFNγ) were increased significantly in the acute stage of KD (Wang et al., 2013). Since lots of cytokines and activated immune cells attacked medium-sized arteries, especially coronary arteries, 20%–25% of untreated patients will develop coronary artery aneurysm (CAA) (Gersony, 2009), which has made KD the leading cause of acquired heart disease among children in developed countries (Kato et al., 1975).

Intravenous immunoglobulin (IVIG) contains pooled immunoglobulin G (IgG) from the plasma of over thousand blood donors and is widely used in people with weakened immune systems or other diseases to fight off infections (Lünemann et al., 2015). High-dose IVIG therapy is effective in resolving inflammation from KD and reducing occurrence of CAA. However, 10%–20% of KD patients will develop IVIG resistance, defined by recrudescent or persistent fever for over 36 h after the end of the IVIG infusion primary therapy. In addition, these patients have persistent inflammation and increased risk of developing CAA (McCrindle et al., 2017). Therefore, uncovering the mechanism of IVIG resistance in KD is urgently needed. While the mechanism of IVIG action is complicated and how it works on KD is still confused and unknown, at present, several studies have shown that genetic polymorphisms, especially some immune functional genes, are associated with IVIG resistance, such as inositol 1.4.5-trisposhate 3-kinase C (*ITPKC*), Fcγ IgG receptor 2A (*FCGR2A*), *CD40*, and interferon-gamma (*IFN*-*γ*) (Onouchi et al., 2008; Khor et al., 2011; Lee et al., 2012; Onouchi et al., 2013; Huang et al., 2016). These studies suggested that genetic factors might be involved in IVIG resistance.

Eukaryotic translation initiation factor 2-alpha kinase 4 (*EIF2AK4*, also known as *GCN2*) is a member of the kinase family that phosphorylates the alpha subunit of eukaryotic translation initiation factor-2 (eIF2a)(Wang et al., 2019). EIF2AK4 phosphorylates eIF2a on the serine 51 site and reduces GDP/GTP exchange activity subsequently. In addition, this resulted in mRNA translation changes and subsequently modulated cellular physiological activities (McGaha et al., 2012).

*EIF2AK4* mutation was found in patients classified as having idiopathic and heritable pulmonary arterial hypertension (Hadinnapola et al., 2017). Histopathology of *EIF2AK4* mutation carriers in pulmonary veno-occlusive disease (PVOD) patients was distinctive from noncarriers regarding arterial remodeling, with significantly more severe intimal fibrosis and less severe medial hypertrophy (Nossent et al., 2018). Furthermore, under nutrient-deprived conditions, *EIF2AK4* could promote angiogensis of endothelial cells by increasing VEGF expression (Longchamp et al., 2018). These literature studies showed that *EIF2AK4* could modulate vascular remodeling and angiogensis, which are closely associated with coronary arterial lesions (CALs) of KD (Takahashi et al., 2013). What’s more, *EIF2AK4* was also found to regulate cytokine production and macrophage function in several infectious diseases. *Eif2ak4* knockout mice challenged with lipopolysaccharide (LPS) showed reduced inflammatory response, including decreased IL-6 and IL-12 expression, as compared to wild-type mice (Liu et al., 2014). Interestingly, IL-6 and IL-10 were at a high level in the IVIG resistant group compared to the IVIG response group after IVIG treatment (Wang et al., 2013). In inflammatory kidney disease, IFN-γ–activated EIF2AK4 could suppress proinflammatory cytokine production in glomeruli and reduce macrophage recruitment to the kidneys (Chaudhary et al., 2015), while Ravindran et al. (2016) showed that EIF2AK4 also controlled intestinal inflammation through inhibiting inflammasome activation and IL-1β production. In conclusion, these studies indicate that *EIF2AK4* may be associated with KD.

Our team has worked on the area of the etiology and therapy effect of KD for many years (Che et al., 2018; Lin et al., 2021; Wang et al., 2021). We found that the single-nucleotide polymorphism (SNP) of immune and/or cardiovascular-related genes were usually related to IVIG therapy outcome of KD, such as *IL-1β* (Fu et al., 2019), *PLA2G7* (Gu et al., 2020), *P2RY12* (Wang et al., 2020), and *MRP4* (Wang et al., 2021). However, evidence regarding the polymorphisms of *EIF2AK4* and IVIG resistance of KD is very scarce. Based on this background, we performed this epidemiology study to investigate whether *EIF2AK4* is related to IVIG resistance of KD by examining the association between *EIF2AK4* polymorphism (rs4594236) and the risk of IVIG resistance of KD.

## Materials and Methods

### Study Subjects

A total of 1,029 KD patients from the Guangzhou Women and Children’s Medical Center between January 2014 and December 2019 were enrolled in this study. All individuals with KD were diagnosed by pediatricians based on the criteria of the American Heart Association (Newburger et al., 2004; McCrindle et al., 2017). IVIG resistance was defined as persistent or recrudescent fever (temperature ≥38.0°C, measured axilla or orally) for over 36 h, but for a period of less than 7 days, after completion of the first IVIG infusion (2 g/kg).

### Polymorphism Genotyping and DNA Extraction

Peripheral blood was collected from KD patients after treatment completion. Genomic DNA was extracted with a TIANamp Blood DNA Kit (DP318, TIANGEN Biotech, Beijing) following the guidance of the manufacturer’s instructions (Wu et al., 2020). Specific fluorescent allele probes for rs4594236 were purchased from ABI (Thermo Fisher Scientific, United States). PCR was performed in 384-well plates with an ABI-Q6 Sequence Detection System machine (Thermo Fisher Scientific).

The genotyping of the SNP was conducted using a TaqMan SNP genotyping assay (Lin et al., 2021). Laboratory technicians were blind to the sample information, including the identities of the replicate aliquots. 10% of the samples from both groups were arbitrarily chosen to repeat the genotyping results. A concordance rate of 100% was obtained.

### Statistical Analysis

Statistical analysis of this study was performed by using SAS software (version 9.4; SAS Institute, Cary, NC). Pearson’s chi square test was used to evaluate the significant differences between IVIG response and IVIG resistant cases in the distribution of demographic variables and genotype frequency. Odds ratios (ORs) and 95% confidence intervals (CIs) were calculated by logistic regression analysis for measuring the association between the *EIF2AK4*/rs4594236 polymorphism and the risk of IVIG treatment resistance in KD patients. Furthermore, stratification analysis was performed, classified by age and gender. We also performed the eQTL analysis using the GTEx Portal web site (https://www.gtexportal.org/home/) to predict potential associations between the SNP and gene-expression levels (Consortium, 2013). A *p*-value of less than 0.05 was regarded as statistically significant.

## Results

### Population Characteristics

The characteristic distribution of 795 IVIG therapy response KD patients and 234 IVIG therapy resistant KD patients is shown in Table 1. The average age of the IVIG response group was 25.14 ± 20.33 months (rang 1–131 months), and it was 26.08 ± 21.80 months (rang 2–132 months) for the IVIG resistant group. 67.17% of the KD patients who were responsive to IVIG therapy were men, and the male ratio was 72.65% in KD patients who were resistant to IVIG therapy. The proportion of women was 32.83% and 27.35%, respectively, while there were no significant difference in age (*p* = 0.3750) and gender (*p* = 0.1096) between the IVIG response group and the resistant group.

**TABLE 1:** Characteristic distribution in the IVIG therapy resistant group and the response group of KD patients.

Variables	IVIG resistance (*n* = 234)		IVIG response (*n* = 795)		*p* ^a^
	No.	%	No.	%	
Total	234	100	795	100
Age range, month	2-132		1-131		
Mean ± SD	26.08 ± 21.80		25.14 ± 20.33		
≤60	219	93.59	756	95.09	0.3750
>60	15	6.41	39	4.91
Gender					
Male	170	72.65	534	67.17	0.1096
Female	64	27.35	261	32.83

a Two-sided *χ* ^2^ test for distributions between KD patients with the IVIG therapy–resistant and –response group.

### Analysis of the Association Between *EIF2AK4/*rs4594236 Polymorphism and Intravenous Immunoglobulin Resistance

The genotype frequency distribution of *EIF2AK4/*rs4594236 polymorphism in the KD IVIG resistant group and response group is described in Table 2. To explore the association between *EIF2AK4/*rs4594236 polymorphism and the risk to IVIG therapy resistance, we performed *χ* ^2^ test analysis. We found that *EIF2AK4/*rs4594236 polymorphism was significantly associated with increased IVIG therapy resistance risk in KD patients (AG vs. AA: adjusted OR = 1.71, 95% confidence interval (CI) = 1.17–2.51, and *p* = 0.0061; GG vs. AA: adjusted OR = 2.09, 95% confidence interval (CI) = 1.36–3.23, and *p* = 0.0009; AG/GG vs. AA: adjusted OR = 1.82, 95% CI = 1.27–2.63, and *p* = 0.0013; GG vs. AG/AA: adjusted OR = 1.45, 95% confidence interval (CI) = 1.04–2.02, and *p* = 0.0306). The results indicated that patients with a GG/AG genotype had a higher risk of suffering IVIG therapy resistance than patients with an AA genotype, suggesting the resistive effect of this SNP against IVIG therapy.

**TABLE 2:** Genotype distribution frequency of *EIF2AK4*/rs4594236 polymorphism in the IVIG therapy–resistant group and –response group of KD patients.

Genotype	IVIG resistance (*N* = 234)	IVIG response (*N* = 795)	*p*-value^a^	OR (95% CI)	*p*-value	Adjusted OR (95% CI)	*p*-value^b^
AA	43 (18.38)	231 (29.06)	0.0020	1		1	
AG	126 (53.85)	397 (49.94)		1.71 (1.16–2.50)	0.0062	1.71 (1.17–2.51)	0.0061
GG	65 (27.78)	167 (21.01)		2.09 (1.36–3.23)	0.0009	2.09 (1.36–3.23)	0.0009
Additive				1.43 (1.16–1.77)	0.0009	1.43 (1.16–1.77)	0.0009
Dominant	191 (81.62)	564 (70.94)	0.0008	1.82 (1.26–2.63)	0.0013	1.82 (1.27–2.63)	0.0013
Recessive	169 (72.22)	628 (78.99)	0.0322	1.45 (1.04–2.02)	0.0299	1.45 (1.04–2.02)	0.0306

a Two-sided *χ* ^2^ test for distributions between the IVIG therapy–resistant group and –response group of KD patients.

b Adjusted for age and gender status in logistic regression models.

### Analysis of the Association Between *EIF2AK4/*rs4594236 Polymorphism and Coronary Arterial Lesions

It is well known that *EIF2AK4* is involved in vascular remodeling (Nossent et al., 2018), which is the critical step for CAL formation. Therefore, the association between *EIF2AK4*/rs4594236 polymorphism and CAL formation was explored. Patients with KD were then divided into the CAL group and the NCAL group depending on whether they had CAL or not, and *EIF2AK4*/rs4594236 genotyping was performed on the two groups (Table 3). As shown in Table 3, *EIF2AK4*/rs4594236 was not associated with CAL formation. We then analyzed the relation between *EIF2AK4*/rs4594236 polymorphism and CAA formation (the serious lesions of the coronary artery) of KD. However, there was no significant association observed between *EIF2AK4*/rs4594236 and CAA (Table 4).

**TABLE 3:** Genotype distribution frequency of *EIF2AK4*/rs4594236 polymorphism in the NCAL group and CAL group of KD patients.

Genotype	CAL (*N* = 408)	NCAL (*N* = 621)	*p*-value^a^	OR (95% CI)	*p*-value	Adjusted OR (95% CI)	*p*-value^b^
AA	107 (26.23)	167 (26.89)	0.8297	1.000		1.000	
AG	212 (51.96)	311 (50.08)		1.064 (0.789–1.435)	0.6846	1.041 (0.767–1.414)	0.7945
GG	89 (21.81)	143 (23.03)		0.971 (0.678–1.391)	0.874	0.941 (0.652–1.358)	0.7447
Additive				0.989 (0.827–1.182)	0.9024	0.973 (0.811–1.168)	0.7686
Dominant	301 (73.77)	454 (73.11)	0.8128	1.035 (0.780–1.373)	0.8132	1.010 (0.756–1.349)	0.9482
Recessive	319 (78.19)	478 (76.97)	0.6481	0.933 (0.691–1.259)	0.65	0.916 (0.674–1.245)	0.5751

a Two-sided *χ* ^2^ tests were used to determine differences in genotype distributions between KD with and without CAL.

b Adjusted for age and gender status in logistic regression models.

**TABLE 4:** Genotype and allele frequencies of *EIF2AK4* in KD Patients with (CAA) or without CAA (NCAA).

Genotype	CAA (*N* = 216)	NCAA (*N* = 813)	*p*-value^a^	OR (95% CI)	*p*-value	Adjusted OR (95% CI)	*p*-value^b^
AA	55 (25.46)	219 (26.94)	0.8108	1.000		1.000	
AG	114 (52.78)	409 (50.31)		1.110 (0.773–1.593)	0.5717	1.088 (0.754–1.571)	0.6506
GG	47 (21.76)	185 (22.76)		1.012 (0.654–1.564)	0.9586	0.983 (0.632–1.530)	0.9389
Additive				1.010 (0.815–1.251)	0.9289	0.995 (0.800–1.237)	0.9648
Dominant	161 (74.54)	594 (73.06)	0.6619	1.079 (0.766–1.521)	0.6631	1.055 (0.745–1.495)	0.7615
Recessive	169 (78.24)	628 (77.24)	0.7548	0.944 (0.657–1.356)	0.7556	0.929 (0.643–1.342)	0.6946

a Two-sided *χ* ^2^ tests were used to determine differences in genotype distributions between KD with and without CAA.

b Adjusted for age and gender status in logistic regression models.

### Stratification Analysis

We further explored the association between *EIF2AK4/*rs4594236 polymorphism and the risk effect of IVIG resistance on certain subgroups classified by age and gender (Table 5). Compared with the rs4594236 AA genotype, the risk effect of the rs4594236 GG/AG genotype was more prominent in male patients of all ages (adjust OR = 1.91, 95% CI = 1.23–2.95, and *p* = 0.0039).

**TABLE 5:** Stratification analysis of *EIF2AK4*/rs4594236 polymorphism in the IVIG therapy resistant group and response group of KD patients.

Variables	rs4594236 (IVIG resistance/IVIG response)		*p*-value^a^	OR (95%CI)	*p*-value	Adjusted OR (95% CI)	Adjust *p*-value^b^
	AA	AG/GG					
Age, months							
≤60	42/216	177/540	0.0044	1.69 (1.16–2.44)	0.0059	1.67 (1.15–2.43)	0.0068
>60	1/15	14/24	0.012	8.75 (1.04–73.54)	0.0458	9.43 (1.08–82.13)	0.0421
Gender							
Male	30/154	140/380	0.0029	1.89 (1.22–2.93)	0.0042	1.91 (1.23–2.95)	0.0039
Female	13/77	51/184	0.1314	1.64 (0.85–3.19)	0.1437	1.62 (0.83–3.15)	0.1551

a Two-sided *χ* ^2^ test for distributions between the IVIG therapy resistant group and response group of KD patients.

b Adjusted for gender/age status in logistic regression models.

### Expression Quantitative Trait Loci Analyses

**FIGURE 1:**
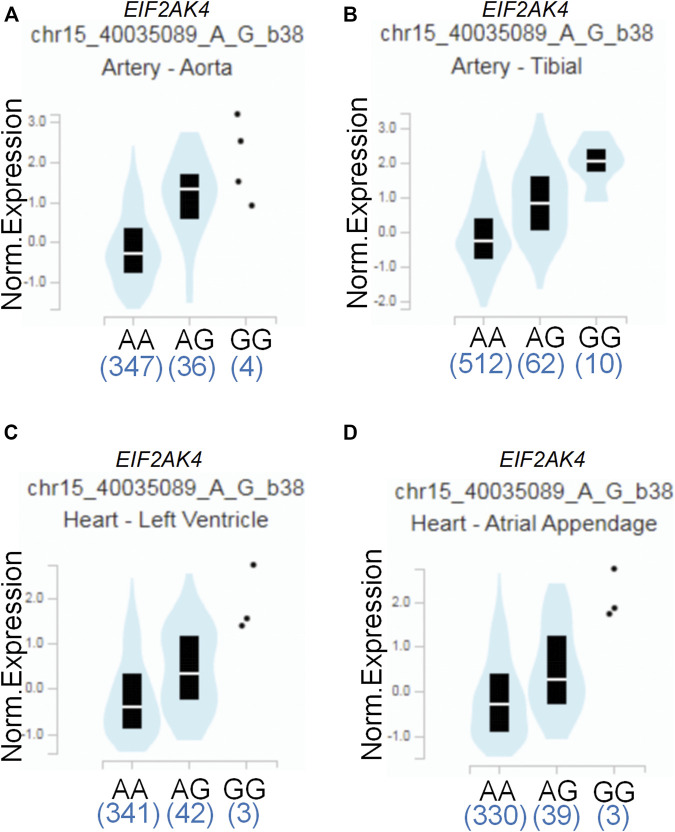
Functional implication of the *EIF2AK4* gene rs4594236 polymorphism in human tissues. **(A)** The genotype of rs4594236 and expression of the *EIF2AK4* gene in the artery of the aorta were searched on the public database GTEx Portal. *p* = 2.6 × 10^−20^. **(B)** The genotype of rs4594236 and expression of the *EIF2AK4* gene in the artery of the tibia were searched on the public database GTEx Portal. *p* = 2.4 × 10^−18^. **(C)** The genotype of rs4594236 and expression of the *EIF2AK4* gene in the left ventricle of the heart were searched on the public database GTEx Portal. *p* = 3.4 × 10^−8^. **(D)** The genotype of rs4594236 and expression of the *EIF2AK4* gene in the atrial appendage of the heart were searched on the public database GTEx Portal. *p* = 2.1 × 10^−8^.

**FIGURE 2:**
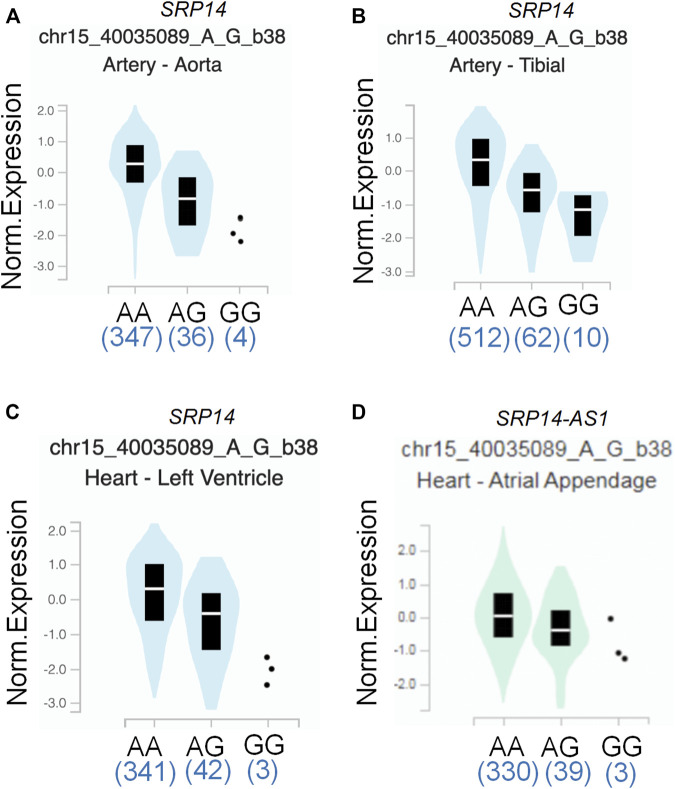
Functional prediction of rs4594236 on a neighboring gene. **(A)** Correlation between rs4594236 and *SRP14* gene expression in the artery of the aorta. *p* = 6.9 × 10^−10^. **(B)** Correlation between rs4594236 and *SRP14* gene expression in the artery of the tibia. *p* = 2.3 × 10^−9^. **(C)** Correlation between rs4594236 and *SRP14* gene expression in the left ventricle of the heart. *p* = 3.4 × 10^−8^. **(D)** Correlation between rs4594236 and *SRP14-AS1* gene expression in the atrial appendage of the heart. *p* = 9.8 × 10^−9^.

To assess the putative functional relevance of rs4594236 polymorphism affecting *EIF2AK4* mRNA expression, we used the data released from Genotype-Tissue Expression (GTEx) website. It was found that individuals carrying the rs4594236 G allele displayed significantly higher *EIF2AK4* mRNA levels in the artery of the aorta and tibia, the atrial appendage, and the left ventricle of the heart than those with the rs4594236 A allele (Figure 1). Furthermore, we evaluated the impact of the rs4594236 polymorphism on the mRNA level of the neighboring genes in the above-mentioned tissues and found that signal recognition particle 14 (SRP14) (or divergent transcript (SRP14-AS1)) mRNA levels in tissues with the rs4594236 G allele were significantly lower than those with the rs4594236 A allele (Figure 2).

## Discussion

IVIG has been the optimal and effective treatment for KD to reduce the prevalence of coronary-artery abnormalities and systemic inflammation until now (Newburger et al., 1986). Although the molecular and cellular basis of IVIG function is complicated and remains unknown, some evidence indicated that genetic factors played an important role in IVIG treatment activities. Taking the fact that several genes were associated with the susceptibility of KD and the rates of IVIG resistant patients differ among different ethnic groups (Kashef et al., 2005; Uehara et al., 2008; Tremoulet et al., 2011), some hot genetic factors, especially immune functional genes, were examined to be associated with IVIG resistance, such as *FcγR2C* and *FcγR3B* (Makowsky et al., 2013). Furthermore, Weng et al. (2010) had found that patients with *IL-1β* (−511 TT) and *IL-1β* (−31 CC) genotypes had increased risk of IVIG resistance and were associated with initial IVIG treatment failure based on a study of 156 KD patients (136 with and 20 without response to IVIG treatment) among Taiwanese children .

Herein, we demonstrated a potentially contributing role of *EIF2AK4*/rs4594236 polymorphism in IVIG resistance in KD and for the first time reported that *EIF2AK4*/rs4594236 polymorphism could predispose to IVIG resistance in southern Chinese KD children.

*EIF2AK4* is a high molecular weight protein kinase activated by uncharged tRNA (Wek et al., 1990; Nakamura et al., 2018; Schmidt et al., 2019). Activated *EIF2AK4* can phosphorylate eIF2a to upregulate ATF4 translation, which in turn increases amino acid biosynthetic and activated transport pathways (Harding et al., 2000; Harding et al., 2003). The physiological functions of *EIF2AK4* currently remain poorly understood, but its function in human diseases has recently been emphasized.

Several studies demonstrated that *EIF2AK4* was associated with vascular remodeling (Lu et al., 2014; Nossent et al., 2018; Chen et al., 2021). One possible mechanism was that *EIF2AK4* dysfunction enhanced collagen I gene transcription via the ATF3/p38 pathway, which led to increased collagen deposition in the pulmonary artery (Chen et al., 2021). As vascular remodeling is critical for CAL formation of KD, we also investigated the association between *EIF2AK4*/rs4594236 polymorphism and CAL or CAA formation of KD. However, we found that *EIF2AK4*/rs4594236 polymorphism was not associated with either CAL or CAA formation. The possible reason may be that the *EIF2AK4* expression level was decreased significantly in *EIF2AK4* mutation PVOD patients who had undergone vascular modeling. While the data from GTEx showed that individuals carrying the rs4594236 G allele displayed significantly higher EIF2AK4 mRNA levels in the artery of the aorta and tibia, the atrial appendage, and the left ventricle of the heart than those with the rs4594236 A allele (Figure 1), it is indicated that the patients with an rs4594236 G allele have a higher risk of IVIG therapy resistance and the EIF2AK4 expression level. In other words, the patients with higher EIF2AK4 expression tend to have a higher IVIG resistant incidence rate. Hence, different expression levels of EIF2AK4 stimulated diverse downstream signals to regulate cell physiological functions differently.

On the other hand, McGaha et al. found loss of *EIF2AK4*-enhanced inflammatory macrophage transcription with a crowd of proinflammatory cytokine expression and production. In addition, the activated regulatory macrophage was attenuated with a decrease in the Arg1 and CCL22 mRNA expression and IL-10 protein level at the same time. Mechanistically, EIF2AK4 altered the myeloid function by activating the CREB-2/ATF4 signal pathway, which was required for maturation and polarization of macrophages in both mice and humans (Halaby et al., 2019). Interestingly, IVIG treatment promotes tumor-associated macrophages from M2 to M1 polarization, and the IVIG effect was dependent on the activation/polarization state of macrophages (Domínguez-Soto et al., 2014). It is possible that the immunomodulatory effect of IVIG observed in other autoimmune diseases such as KD follows a similar pattern. In addition, our results showed the IVIG resistant risk of KD patients may be linked to the upregulated expression levels of the *EIF2AK4* gene (Figure 1); thus, we deduced the hypothesis that IVIG treatment promoted macrophage M1 polarization while the immunomodulatory function of the M1 macrophage was inhibited by upregulated EIF2AK4, which caused persistent inflammation. However, more functional experiments need to be carried out to support the possible mechanism.

To date, studies have been conducted regarding the epidemiologic assessment of *EIF2AK4* gene SNPs. Deng et al. carried out a genome-wide association study of body mass index (BMI) from a cohort containing 597 northern Chinese patients and reported 281,533 SNPs. They found that two adjacent SNPs (rs4432245 and rs711906) of *EIF2AK4* were significantly associated with BMI (Yang et al., 2014). Given the critical role of *EIF2AK4* in immunity reactions, it is necessary to investigate the association between *EIF2AK4* gene SNPs and IVIG resistance of KD. The current study revealed that the SNP rs4594236 polymorphism in the *EIF2AK4* gene was associated with increased risk to IVIG resistance of KD in southern Chinese population. Compared with the rs4594236 AA genotype, the AG/GG genotype increased the IVIG resistant risk significantly, especially in male KD patients. We then explored the potential mechanism for the risk role of rs4594236 polymorphism in IVIG resistance. The results from eQTLs analysis indicated that IVIG resistant risk of KD was associated with upregulated expression levels of the *EIF2AK4* gene (Figure 1). We also evaluated the impact of rs4594236 polymorphism on the mRNA level of the neighboring genes. We found that the *SRP14* (or *SRP14-AS1*) mRNA level with the rs4594236 G genotype was significantly lower than that in cells with the rs4594236 A genotype (Figure 2). *SRP14* is a universal ribonucleoprotein, and combined with SRP9 as a heterodimer, it can recognize the RNA UGUNR motif to regulate target gene translation (Bovia et al., 1995; Hasler and Strub, 2006). Thus, we hypothesized that SRP14 combined with the specific RNA motif of *EIF2AK4* and negatively regulated the *EIF2AK4* mRNA translation level to trigger downstream physiological reactions to inhibit IVIG therapy response.

The major strength of this study is its novelty; as we know, this is the first study that focuses on the *EIF2AK4* function in IVIG therapy resistance of KD at present. However, our study still has some limitations. First, the enrolled patients of this study were mainly from the southern Chinese population; the study needs multicenter subjects from other geographic populations to support and evaluate the applicability of the findings to other ethnicities. Second, only one functional SNP in the *EIF2AK4* gene was included in this study; more potentially functional SNPs of *EIF2AK4* need to be investigated in the future. Last but not least, the exact biological mechanism of *EIF2AK4* in IVIG resistance of KD is worthy of further investigation.

## Data Availability

The raw data supporting the conclusions of this article will be made available by the authors, without undue reservation.
