# Supplementary material for: The EIF2AK4/rs4594236 AG/GG Genotype Is a Hazard Factor of Immunoglobulin Therapy Resistance in Southern Chinese Kawasaki Disease Patients
Source: Front Genet. 2022 Jun 22;13:868159. doi: 10.3389/fgene.2022.868159 (PMC9257007; doi:10.3389/fgene.2022.868159)
Supplement: Supplementary file 1 [file DataSheet1.DOCX]

The detail *EIF2AK4/*rs4594236 was list below:

| **Gene : Consequence** | EIF2AK4 : 3 Prime UTR Variant |
| --- | --- |
| **Position** | chr15:40035089 (GRCh38.p13) |
| **Alleles** | A>G |
| **Frequency** | A=0.513, G=0.487(East Asian) |
